# Human CD56+ Cytotoxic Lung Lymphocytes Kill Autologous Lung Cells in Chronic Obstructive Pulmonary Disease

**DOI:** 10.1371/journal.pone.0103840

**Published:** 2014-07-31

**Authors:** Christine M. Freeman, Valerie R. Stolberg, Sean Crudgington, Fernando J. Martinez, MeiLan K. Han, Stephen W. Chensue, Douglas A. Arenberg, Catherine A. Meldrum, Lisa McCloskey, Jeffrey L. Curtis

**Affiliations:** 1 Research Service, VA Ann Arbor Healthcare System, Ann Arbor, Michigan, United States of America; 2 Pulmonary & Critical Care Medicine Division, Department of Internal Medicine, University of Michigan Health System, Ann Arbor, Michigan, United States of America; 3 Graduate Program in Immunology, University of Michigan, Ann Arbor, Michigan, United States of America; 4 Pathology & Laboratory Medicine Service, VA Ann Arbor Healthcare System, Ann Arbor, Michigan, United States of America; 5 Department of Pathology, University of Michigan Health System, Ann Arbor, Michigan, United States of America; 6 Pulmonary & Critical Care Medicine Section, Medicine Service, VA Ann Arbor Healthcare System, Ann Arbor, Michigan, United States of America; Beth Israel Deaconess Medical Center, Harvard Medical School, United States of America

## Abstract

CD56+ natural killer (NK) and CD56+ T cells, from sputum or bronchoalveolar lavage of subjects with chronic obstructive pulmonary disease (COPD) are more cytotoxic to highly susceptible NK targets than those from control subjects. Whether the same is true in lung parenchyma, and if NK activity actually contributes to emphysema progression are unknown. To address these questions, we performed two types of experiments on lung tissue from clinically-indicated resections (*n* = 60). First, we used flow cytometry on fresh single-cell suspension to measure expression of cell-surface molecules (CD56, CD16, CD8, NKG2D and NKp44) on lung lymphocytes and of the 6D4 epitope common to MICA and MICB on lung epithelial (CD326+) cells. Second, we sequentially isolated CD56+, CD8+ and CD4+ lung lymphocytes, co-cultured each with autologous lung target cells, then determined apoptosis of individual target cells using Annexin-V and 7-AAD staining. Lung NK cells (CD56+ CD3−) and CD56+ T cells (CD56+ CD3+) were present in a range of frequencies that did not differ significantly between smokers without COPD and subjects with COPD. Lung NK cells had a predominantly “cytotoxic” CD56+ CD16+ phenotype; their co-expression of CD8 was common, but the percentage expressing CD8 fell as FEV_1_ % predicted decreased. Greater expression by autologous lung epithelial cells of the NKG2D ligands, MICA/MICB, but not expression by lung CD56+ cells of the activating receptor NKG2D, correlated inversely with FEV_1_ % predicted. Lung CD56+ lymphocytes, but not CD4+ or CD8+ conventional lung T cells, rapidly killed autologous lung cells without additional stimulation. Such natural cytotoxicity was increased in subjects with severe COPD and was unexplained in multiple regression analysis by age or cancer as indication for surgery. These data show that as spirometry worsens in COPD, CD56+ lung lymphocytes exhibit spontaneous cytotoxicity of autologous structural lung cells, supporting their potential role in emphysema progression.

**Trial Registration:**

ClinicalTrials.gov NCT00281229

## Introduction

Chronic obstructive pulmonary disease (COPD), currently the 3^rd^ leading cause of death in the United States [1], is a chronic disease that is associated with irreversible parenchymal lung destruction, airways remodeling, mucus hypersecretion, and infiltration by inflammatory cells [2], [3]. COPD is believed to result from inhaled oxidants, including indoor air pollution from biomass fuels and exposure to cigarette smoke. However, for unknown reason, only a minority of smokers develop COPD. In smokers who do develop COPD, the chronic inflammatory response in the airways and parenchyma is a result of both innate and adaptive immune responses. Developing therapies to combat progression of emphysema is particularly important, as this phenotype of COPD is associated with increased mortality [4] that is not altered by current therapies, other than lung volume-reduction surgery in selected patients [5],[6].

Natural killer (NK) cells and CD56-expressing T cells are cytotoxic lymphocytes that play roles in autoimmunity and chronic infection that are crucial, although not always ultimately beneficial to the host. These two cell types share a “poised effector state”, i.e., the ability to lyse abnormal cells and to secrete cytokines and chemokines rapidly [7]. Both also express the CD56 surface antigen. They are distinguished from each other by surface expression of αβ TCR and associated CD3 signaling complexes by CD56+ T cells, but not by NK cells. Both NK cells and CD56+ T cells have been implicated in COPD pathogenesis based on data from both human subjects and experimental animal models. NK cells and CD56+ T cells from sputum and bronchoalveolar lavage (BAL) of COPD subjects were proportionally increased, relative to healthy smokers or non-smoking healthy subjects [8]. In COPD subjects, NK cells and CD56+ T cells in sputum and BAL also showed greater in vitro killing [8], [9]. In murine cigarette smoke (CS) exposure models, we and others have shown that NK cells from CS-exposed mice are increased in the lungs, display an activated, or “primed” phenotype [10], [11], and are more cytotoxic towards target cells [12]. Because human and animal studies have strongly implicated increased apoptosis of lung structural cells in emphysema pathogenesis [13]–[15], defining whether there are roles for NK cell or CD56+ T cells in the disease process is an important objective.

Commitment of an NK cell to kill an individual target cell is tightly regulated by the balance between inhibitory and activating signals received via a multitude of NK cell surface receptors [16]. Inhibitory NK receptors recognize MHC Class I molecules, which are expressed on healthy cells, protecting them from NK-mediated killing, but often lost on viral transformation [17]. Conversely, activating NK receptors, including the natural killer group 2/member D (NKG2D) and natural cytotoxicity receptors (NCRs) such as NKp44, recognize glycoproteins expressed both by pathogens and by damaged host cells. In particular, NKG2D binds multiple ligands absent from healthy cells but induced by infection, transformation, DNA damage and oxidative stress, including the MHC class I chain-related genes A and B (MICA & MICB) [18]. Expression of NKG2D ligands by lung epithelial cells has also been shown for human cells in vitro, in a murine CS model and in lung tissue from COPD patients [19], [20]. Mice deficient in NKG2D exhibit attenuated airspace enlargement in a model of CS-induced emphysema [21].

Functional characteristics of NK cells differ depending on the environment from which they are isolated [22]. For instance, murine lung NK cells are phenotypically more mature but less cytotoxic than splenic NK cells [23]. In contrast to data from BAL and sputum, the cytotoxic effector function of peripheral blood NK cells in COPD subjects is reduced compared to controls [24], [25]. Therefore, one goal of the current study was to define the phenotype and cytotoxic ability of NK cells and CD56+ T cells from human lung parenchyma, which should be more relevant to emphysema pathogenesis than cells in the alveolar spaces or sputum. A second important goal was to determine whether human lung NK cells and CD56+ T cells could exhibit natural cytotoxicity against lung parenchymal cells from the same (“autologous”) individual. Our results provide the novel evidence that NK activity could contribute to emphysema pathogenesis in humans.

## Methods and Materials

### Ethics statement

Studies and consent procedures were performed in accordance with the Declaration of Helsinki at the VA Ann Arbor Healthcare System and the University of Michigan Health System and were approved by the Institutional Review Board at each site (FWA 00000348 and FWA 00004969, respectively). Written informed consent was obtained preoperatively.

### Specimens and patient population

We recruited and consented subjects undergoing clinically-indicated resections for pulmonary nodules, lung volume reduction surgery, or lung transplantation. Only non-neoplastic lung tissue remote from the nodules and lacking post-obstructive changes as judged by a Pathologist was collected. Because not all experiments can be performed on every lung sample, two cohorts of subjects were used to complete these experiments (Table 1). Cohort A (*n* = 35) was used for both flow cytometry and cell isolations. Cohort B (*n* = 25) was used exclusively for flow cytometric analyses. All subjects (*n* = 60) underwent preoperative spirometry, prospectively collected medication history and clinical evaluation by a pulmonologist. We categorized subjects using the 2008 classification of the Global Initiative for Chronic Obstructive Lung Disease (GOLD) [26]. Subjects (*n* = 16) with a smoking history of 10 pack years or greater, a ratio of forced expiratory volume in 1 second to forced vital capacity (FEV_1_/FVC) >0.70, normal spirometry, and no clinical diagnosis of COPD represent smoking controls (Smoker). Subjects (*n* = 19) with a smoking history, FEV_1_/FVC <0.7 and FEV_1_ % predicted ≥50% were considered to have mild COPD (Mild COPD). Subjects (*n* = 25) with a smoking history, FEV_1_/FVC <0.7 and FEV_1_ % predicted <50% were considered to have severe COPD (Severe COPD). Table 1 shows the male-to-female ratio, age range, smoking history, FEV_1_ % predicted, diffusing capacity of the lung for carbon monoxide (DLCO) % predicted, inhaled corticosteroid (ICS) usage, and the indication for lung resection for smokers, mild COPD, and severe COPD subjects for both Cohort A and Cohort B.

**Table 1:** Summary of subject demographics, smoking history, and spirometry.

Cohort A for Figures 1, 2, 4, 5, and Table 2				
Group	Smoker	Mild COPD	Severe COPD	*p* value
Subjects, n	6	14	15	.
Sex ratio, M/F	3/3	13/1	8/7	0.04
Age, years (SD)	67 (9)	66 (8)	60 (8)	0.11
Smoking, pack-years (SD)	73 (54)	61 (32)	59 (28)	0.96
Smoking status (Active/Former)	0/6	8/6	1/14	0.003
FEV_1_, % pred (SD)	92 (12)	77 (18)	26 (11)	<0.0001
DLCO, % pred (SD)	77 (8)	73 (25)	32 (15)	0.0002
ICS usage (yes/no)	1/5	5/9	13/2	0.003
Resection for cancer (yes/no)	6/0	13/1	0/15	<0.0001
**Cohort B for ** **Figure 3**				
**Group**	**Smoker**	**Mild COPD**	**Severe COPD**	***p*** ** value**
Subjects, n	10	5	10	.
Sex ratio, M/F	7/3	3/2	4/6	0.41
Age, years (SD)	59 (8)	72 (6)	59 (7)	0.02
Smoking, pack-years (SD)	52 (24)	81 (69)	58 (21)	0.87
Smoking status (Active/Former)	4/6	2/3	2/8	0.51
FEV_1_, % pred (SD)	89 (16)	71 (20)	23 (9)	<0.0001
DLCO, % pred (SD)	76 (13)	64 (15)	40 (31)	0.11
ICS usage (yes/no)	1/9	3/2	8/2	0.008
Resection for cancer (yes/no)	10/0	5/0	1/9	<0.0001

Data are presented as average (SD), except for sex, smoking status,

ICS, and resection for cancer ratios.

ICS, inhaled corticosteroids;

M, male;

F, female.

### Sample preparation and flow cytometric analysis

Lung samples weighing approximately 3 g were dispersed using a Waring blender without enzyme treatments, which we have previously shown produces single cell suspensions of high viability and functional capacity [27]–[29]. For flow cytometry, cells were filtered through a 30 µm strainer to remove debris and were resuspended in staining buffer (2% Fetal Bovine Serum in Phosphate Buffered Saline). Cells were added in a volume of 100 µl to each flow tube. We used monoclonal antibodies, directly conjugated to a variety of fluorochromes, against the following antigens (clones shown in parentheses): CD45 (HI30), CD3 (UCHT1), CD56 (MEM-188), CD8 (OKT8), NKG2D (1D11), CD326 (9C4), MICA/MICB (6D4), NKp44 (P44-8) (Biolegend, San Diego, CA), and CD16 (3G8) (Life Technologies, Grand Island, NY). Appropriate isotype-matched controls were used in all experiments. Cells were incubated in the dark with primary antibodies for 25 minutes at room temperature, followed by washing and fixation in 2% paraformaldehyde. Cells were analyzed on an LSR II flow cytometer (BD Bioscience, San Jose, CA) equipped with 488 nm blue, 405 nm violet, 561 nm yellow-green, and 640 nm red lasers; details of instrument setup have been described recently [30]. Data were collected using FACS Diva software with automatic compensation, and were analyzed using FlowJo software (Tree Star, Inc., Ashland, OR). A minimum of 20,000 CD45+ events was collected per sample.

### Cell isolation

In separate experiments, viable cells were isolated from the mechanically disaggregated human lung tissue using MACS technology (Miltenyi Biotec, Auburn, CA). Unlabelled lung homogenates were first passed through a MACS LS column in order to remove large cells, such as macrophages. Then cells were incubated with CD56 microbeads for 15 minutes at 4°C. CD56+ cells were positively selected using MACS LS columns and CD56-depleted cells were used to isolate CD8+ cells in a similar fashion. Following CD8+ isolation, CD8− depleted cells were used to isolate CD4+ cells. Cells that were depleted of CD56, CD8, and CD4 were used as target cells in cytotoxicity assays.

### Cytotoxicity assay

Viable target and effector cells (CD56+, CD8+ and CD4+) were counted and resuspended in lymphocyte culture media (10% FBS, 1 mM sodium pyruvate, 0.5 mM 2-Mercaptoethanol, 1 mM HEPES, 100 u/ml penicillin, 100 u/ml streptomycin, 0.292 mg/ml L-Glutamine in RPMI). Target cells were cultured in four separate conditions: with media alone, with CD56+ cells, with CD8+ cells, and with CD4+ cells. Cells were cultured in 96-well plates at a ratio of 10 effector cells (50,000 cells) to 1 target cell (5,000 cells). Plates were briefly centrifuged and then cultured at 37°C and 5% CO_2_ for 4 hours. Cells were then collected, washed, and resuspended in 100 µl of flow cytometry staining buffer. Monoclonal antibodies against CD45 were added and cells were incubated in the dark, with shaking, at room temperature for 25 minutes. Cells were washed with 2 mL of staining buffer. Cells were then stained with the Annexin V and 7-AAD Apoptosis Detection Kit (BD Biosciences, San Jose, CA) according to manufacturer’s instructions. Cells were analyzed immediately by flow cytometry as described above. The percent of cytotoxic cells was determined by the following equation: ((Target alone) – (Target + Effector))/Target alone)*100.

### Statistics

The majority of statistical analyses were performed using GraphPad Prism 6.0 (GraphPad Software, Inc., La Jolla, CA) on a Macintosh Quad-Core Intel Xeon computer running OS X 10.9.2 (Apple; Cupertino, CA). Kruskal-Wallis tests were used to look for significant differences between groups. The unpaired t-test or the Mann-Whitney t-test was used to compare two groups. We used nonparametric (Spearman) correlation analysis to determine the correlation coefficient, *R_S_*. Multiple linear regression was performed using SPSS Statistics 21.0 (IBM Corp.; Armonk, NY). A two-tailed *p* value of <0.05 was considered to indicate significance.

## Results

### Identification and frequency of NK cells and CD56+ T cells in human lung tissue

**Figure 1:**
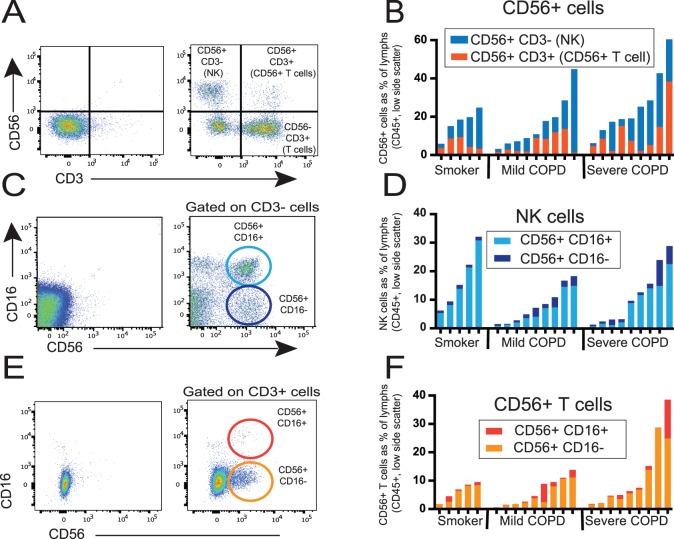
Identification and characterization of human lung NK cells and CD56+ T cells. Lung tissue was dispersed, stained with monoclonal antibodies against CD45, CD3, CD56, and CD16 and analyzed by flow cytometry to select a viable population comprised predominately of lung lymphocytes (CD45+, low side-scatter cells). (**A, C, E**) Representative staining: isotype controls on left, specific staining on right; (**B, D, F**) Frequency of various lung lymphocyte populations in individual subjects as a percentage of the total viable lung lymphocyte population; note difference in scale of panel B. (**A**) Ungated staining for CD3 and CD56 identifies four distinct populations: NK cells (CD56+ CD3−); CD56+ T cells (CD56+ CD3+); conventional T cells (CD56− CD3+); and double-negative cells (predominately B cells). (**B**) NK cells (blue bars) versus CD56+ T cells (orange bars). (**C, D**) After gating on CD3− cells, staining for CD56 and CD16 identifies two lung NK populations: CD56+ CD16+ (light blue circle & columns) and CD56+ CD16− (dark blue circle & columns). (**E, F**) After gating on CD3+ cells, staining for CD56 and CD16 identifies two lung CD56+ T cell populations: CD56+ CD16+ (dark orange circle & columns) and CD56+ CD16− (light orange circle & columns). By Kruskal-Wallis one-way ANOVA, there are no significant differences between subject groups for any of these three lung cell populations (B, D, F).

To determine the frequency of NK cells and CD56+ T cell populations in the human lung, we performed flow cytometry on 23 subjects from Cohort A (**Table 1**). We first gated on live, CD45+ cells. The number of live CD45+ events per subject ranged from 20,000 to 250,000 (mean ± standard deviation (SD) = 100,043±69,529), suggesting that we had a sufficient number of cells for analysis. Next, we gated on low side scatter lymphocytes and then used CD3 and CD56 to identify NK cells (CD56+ CD3−), CD56+ T cells (CD56+ CD3+) and conventional T cells (CD56− CD3+) (**Fig. 1A**). On average, the frequency of the NK cells was higher than the frequency of CD56+ T cells (12.4±10.7% versus 7.8±8.0%, respectively), which agrees with published studies [31], [32]; however in some individuals there were more CD56+ T cells than NK cells (**Fig. 1B**). Overall, we did not see any differences in the frequency of either NK cells or CD56+ T cells between subjects with normal pulmonary function (smokers), subjects with mild COPD, or subjects with severe COPD (*p* = 0.60 for NK cells and *p* = 0.24 for CD56+ T cells).

Human NK cell populations can be separated by the presence and density of CD56 and CD16 (FcγRIII) surface molecules. Two major subsets of human NK cells include CD56^dim^ CD16+ NK cells and CD56^bright^ CD16− NK cells. After identifying NK cells, we analyzed expression of CD16 in combination with CD56 (**Fig. 1C**). Although lung NK cells showed uniformly bright CD56 expression, as anticipated for NK cells outside the peripheral blood, they were very clearly either CD16+ or CD16−. The majority of the NK cells had a CD56+ CD16+ phenotype (**Fig. 1D**) and there was no difference between smokers, subjects with mild COPD, or subjects with severe COPD (*p* = 0.18 for CD56+ CD16+ NK cells and *p* = 0.80 for CD56+ CD16− NK cells). CD56+ T cells have also been shown to express NK cell markers, including CD16, in some settings [33], so we analyzed expression of CD16 versus CD56 on the CD56+ T cell subset (**Fig. 1E**). In contrast to the NK cells, the majority of lung CD56+ T cells did not express CD16 (**Fig. 1F**). We saw no difference in the frequency of either of the two CD56+ T cell subsets between subject groups (*p* = 0.80 for CD56+ CD16− CD56+ T cells and *p* = 0.72 for CD56+ CD16+ CD56+ T cells).

### Decreasing CD8 expression on the NK cell subset correlates with COPD severity

**Figure 2:**
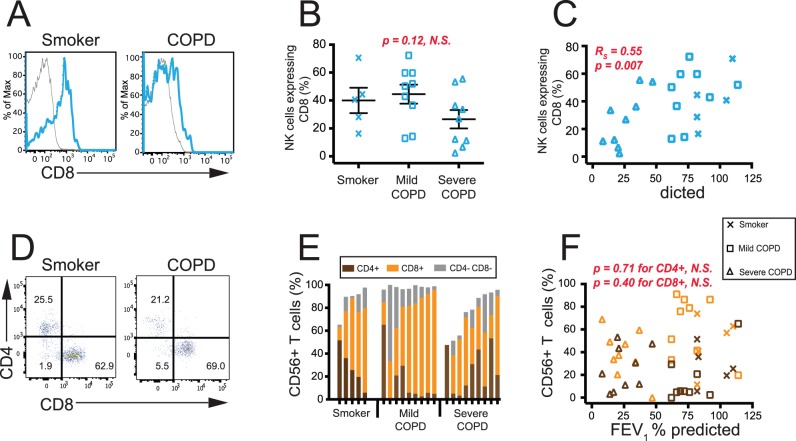
The frequency of lung NK cells, but not lung CD56+ T cells, co-expressing CD8 correlates with FEV_1_ % predicted. (**A–C**) NK cells; (**D–F**) CD56+ T cells. (**A**) Representative staining for CD8 on CD56+ CD3− NK cells from a smoker without COPD (left panel) and a COPD subject (right panel); blue line, CD8+ staining; grey line, isotype control. (**B**) Subjects were categorized by pulmonary function (x-axis) versus the percentage of NK cells that co-express CD8 (y-axis); x, smokers without COPD (*n* = 5); □, mild COPD (*n* = 9); Δ, severe COPD (*n* = 9); Kruskal-Wallis one-way ANOVA testing was used to determine significance. (**C**) The percentage of NK cells that co-express CD8 (y-axis) versus FEV_1_ % predicted (x-axis). Spearman correlation was used to determine the p-value. (**D**) Representative staining for CD8 and CD4 on CD56+ CD3+ T cells from a smoker without COPD (left panel) and a COPD subject (right panel). The numbers in the quadrants are the % of each subset among all lung CD56+ T cells. (**E**) CD4 single-positive (brown bars), CD8 single-positive (orange bars), and CD8/CD4 double-negative (grey bars) are shown as the percentage of lung CD56+ T cells for individual subjects. No difference was seen between groups. (**F**) The percentage of lung CD56+ T cells (y-axis) that express CD4 (brown symbols) or CD8 (orange symbols) versus FEV_1_ % predicted (x-axis); x, smokers without COPD (*n* = 5); □, mild COPD (*n* = 9); Δ, severe COPD (*n* = 9).

We also analyzed expression of CD8 on the lung NK cells from the same subjects. Human peripheral blood NK cells have been shown to co-express CD8 [34], [35], but its expression on human lung NK cells was unknown. Representative histograms show that we were able to detect significant expression of CD8 on NK cells from both healthy smokers and COPD subjects (**Fig. 2A**). There was no significant difference in the percent of NK cells expressing CD8 when we compared subjects with normal pulmonary function to those with mild or severe COPD (**Fig. 2B**). However, the expression of CD8 correlated significantly with FEV_1_ % predicted (**Fig. 2C**), and hence decreased with worsening COPD severity, defined spirometrically. Given the high percentage of NK cells expressing the CD8 surface receptor (39±21%, average of all 23 subjects), studies using single-color staining to study CD8+ T cells would be inadvertently studying NK cells as well. There was no co-expression of CD4 by lung NK cells (not shown).

Similarly, we examined the lung CD56+ T cell population to determine whether they were CD8 single-positive, CD4 single-positive, or CD8, CD4 double-negative (**Fig. 2D**). In most individuals, the majority of lung CD56+ T cells were CD8 single-positive (50.4±25.3%), as has recently been shown for CD56+ T cells in bronchoalveolar lavage [36] . There were no differences in the frequency of these three subsets of lung CD56+ T cells between groups of subjects (**Fig. 2E**) and no relationship of CD8 or CD4 co-expression with FEV_1_ % predicted (**Fig. 2F**).

### Increased percentage of human lung epithelial cells expressing MICA/MICB correlates with severe COPD

**Figure 3:**
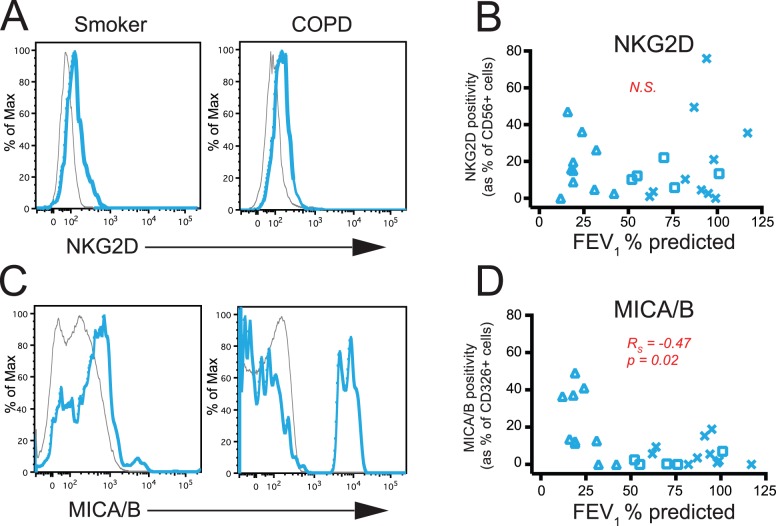
The percentage of epithelial cells expressing MICA/MICB is increased with COPD severity. Human lung tissue was dispersed and stained with monoclonal antibodies against CD45, CD56, NKG2D, CD326, and MICA/MICB. (**A**) Representative staining showing the expression of NKG2D on CD45+ CD56+ cells from a smoker without COPD (left panel) and a subject with COPD (right panel). Blue line, NKG2D+ staining; grey line, isotype control. (**B**) The percentage of CD56+ cells that express NKG2D (y-axis) versus FEV_1_ % predicted (x-axis). x, smokers without COPD (*n* = 10); □, mild COPD (*n* = 5); Δ, severe COPD (*n* = 10). N.S., not significant. (**C**) Representative staining showing the expression of MICA/MICB on CD45−, CD326 (EpCAM)+ epithelial cells from a smoker without COPD (left panel) and a subject with COPD (right panel). Blue line, MICA/MICB+ staining; grey line, isotype control. (**D**) The percentage of CD326+ epithelial cells that express MICA/MICB (y-axis) versus FEV_1_ % predicted (x-axis). x, smokers without COPD (*n* = 10); □, mild COPD (*n* = 5); Δ, severe COPD (*n* = 10). Spearman correlation was used to determine the p value.

In a separate cohort of 25 subjects (**cohort B, described in** **Table 1**), we used flow cytometry to analyze the expression of the activation receptors NKG2D and NKp44, which are both expressed by NK cells. We gated on viable, CD45+, low side-scatter, CD56+ cells, which should entirely contain both NK cell and CD56+ T cell populations. NKG2D was expressed on CD56+ cells from both smokers with normal pulmonary function and COPD subjects (**Fig. 3A**). No difference in the percentage of CD56+ cells expressing NKG2D was observed when the subjects were stratified by FEV_1_ % predicted (**Fig. 3B**) or when subjects were analyzed categorically by COPD status (healthy smokers, *n* = 10; subjects with mild COPD, *n* = 5; subjects with severe COPD, *n* = 10; data not shown), which agrees with data from Borchers et al. [20]. Similarly, no differences were detected between subject groups in the mean fluorescent intensity (MFI) of NKG2D (data not shown). There was also no correlation between receptor expression and other clinical variables (ICS use, surgical indication, pack years, age, DLCO % predicted, and current versus former smoking status). Importantly, we were unable to detect expression of NKp44 on CD56+ cells from the same subjects.

We also used flow cytometry to analyze expression of the NKG2D ligands MICA and MICB on CD45− CD326+ (EpCAM) epithelial cells from the same subjects. We used clone 6D4, which reacts with a common epitope on both MICA and MICB. After gating on our cell population of interest, we were able to detect MICA/MICB on lung epithelial cells from both smokers with normal pulmonary function and subjects with COPD (**Fig. 3C**). The percentage of epithelial cells expressing MICA/MICB inversely correlated with FEV_1_ % predicted (**Fig. 3D**). We did not see any relationship between MFI of MICA/MICB and FEV_1_ % predicted (data not shown).

### Human lung CD56+ lymphocytes can kill autologous CD45− lung cells

To determine whether the lung CD56+ cells were cytotoxic, we next assayed their ability to induce apoptosis of autologous parenchymal target cells from the human lung tissue specimens when co-cultured without additional stimulation. As described in the Methods, single-cell lung tissue suspensions were first depleted of macrophages and then incubated with microbeads against CD56, CD8, and CD4 in sequential steps in order to isolate effector cells (CD56+, CD8+, and CD4+). The remaining autologous lung cells (depleted of macrophages, CD56+, CD8+, and CD4+ cells) were used as target cells. The target cells were then cultured for 4 hours either by themselves or with one of the effector populations at a ratio of 1 target to 10 effectors. All cells from the cultures were collected for immediate analysis using flow cytometry. We identified target cells as CD45− with a high side scatter, as has been shown for lung bronchial epithelial cells [37]. Annexin-V and 7-AAD were used to determine apoptosis of the target cells.

**Figure 4:**
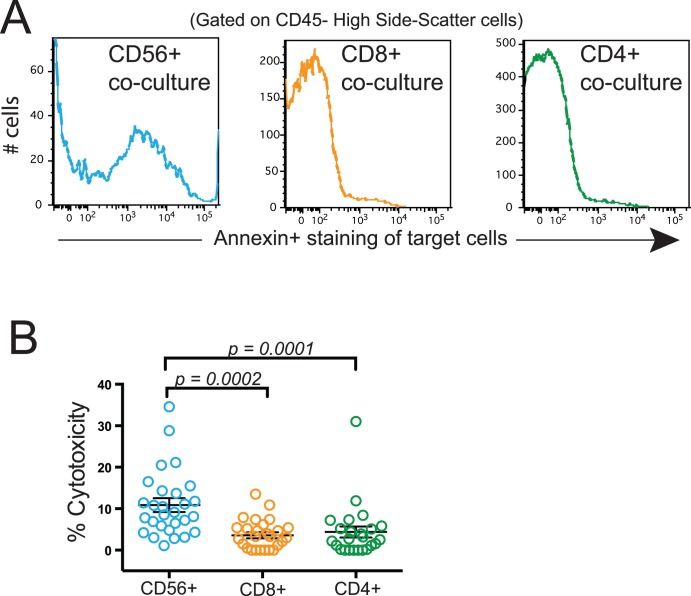
Human lung CD56+ cells spontaneously kill autologous lung CD45− cells in vitro. CD56+ cells were isolated from dispersed human lung tissue using magnetic beads. CD8+ cells were isolated from the CD56 depleted fraction and CD4+ cells were isolated from the CD56− and CD8− depleted fraction. The remaining cells were used as autologous target cells. Target cells were cultured either alone or with CD56+ cells, CD8+ cells, or CD4+ cells at a ratio of 1 target to 10 effectors. After 4 hours, all cells were collected and stained with CD45, Annexin-V, and 7-AAD for flow cytometry. Target cells were identified as CD45− with a high side scatter. (**A**) Representative staining of Annexin-V on target cells that were cultured with CD56+ cells (left panel), CD8+ cells (middle panel), and CD4+ cells (right panel). (**B**) % Cytotoxicity (y-axis) for target cells cultured with CD56+ cells (blue circles), CD8+ cells (orange circles), or CD4+ cells (green circles); *n* = 28. Lines represent the mean ± SEM. The Kruskal-Wallis one-way ANOVA with Dunn’s multiple comparison test was used to determine significant differences between groups.

Lung tissues from subjects in cohort A (*n* = 28) were used in simultaneous paired experiments to compare the ability of CD56+, CD8+, and CD4+ cells from the same subjects to kill autologous parenchymal cells. As shown by the representative staining (**Fig. 4A**), target cells co-cultured with CD56+ cells showed an increase in Annexin-V+ staining, indicative of early apoptosis. There were very few 7-AAD+ (late apoptotic) cells in any of the conditions (data not shown). Analysis of the data from all 28 subjects shows a very significant increase in the ability of lung CD56+ cells to kill autologous target cells (*p* = 0.0002 and 0.0001 compared to lung CD8+ CD56− and lung CD4+ CD56− cells, respectively; **Fig. 4B**). On average, the percentage of specific killing of target cells cultured with CD56+ cells was 10.8±7.8% (mean ± SEM) compared to 3.8±3.4% for CD8+ cells and 4.2±6.4% for CD4+ cells. Target cells that were cultured by themselves showed very little spontaneous death, typically less than 4% (data not shown), but as described in the Methods section, percent specific cytotoxicity was calculated based on the change from this reference population.

### Human lung CD56+ cells from COPD subjects are more cytotoxic than lung CD56+ lymphocytes from subjects with normal pulmonary function

**Figure 5:**
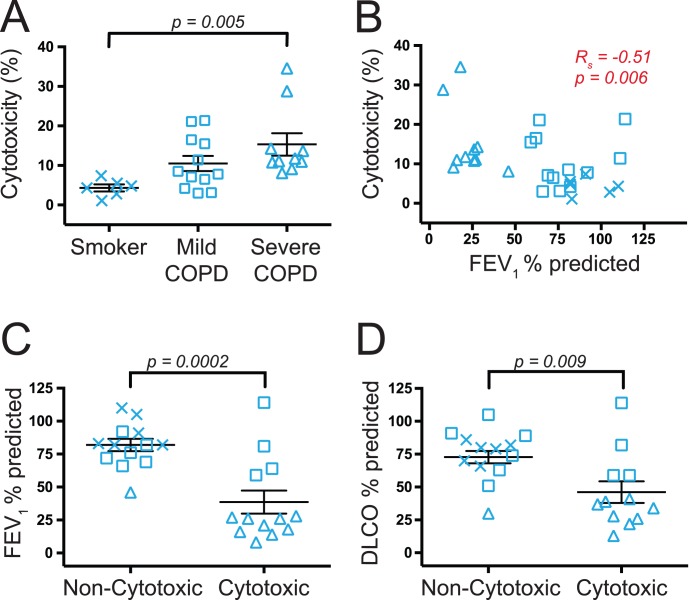
Increased cytotoxicity by lung CD56+ cells correlates with decreased pulmonary function. Human lung CD56+ cells were co-cultured with autologous lung target cells and % cytotoxicity was determined as described in the Methods. (**A**) Subjects were categorized by pulmonary function (x-axis) versus % cytotoxicity (y-axis). The Kruskal-Wallis one-way ANOVA with Dunn’s multiple comparison test was used to determine significant differences between groups. (**B**) FEV_1_ % predicted (x-axis) versus % cytotoxicity (y-axis). Spearman non-parametric correlation was used to determine the p-value. (**C, D**) The same subjects were separated into two groups based on their CD56+ cell function: non-cytotoxic (<8.5% cytotoxicity) or cytotoxic (≥8.5% cytotoxicity) and then FEV_1_ % predicted (**C**) and DLCO % predicted (**D**) were analyzed. The parametric unpaired Student t-test was used to determine significant differences between the two groups. In all figures, x, smokers without COPD (*n* = 6); □, mild COPD (*n* = 12); Δ, severe COPD (*n* = 10). Lines represent the mean ± SEM.

Having established that lung CD56+ cells (which include both NK cells and CD56+ T cells) are able to kill autologous lung target cells, we then separated the subjects into smokers with normal pulmonary function (*n* = 6), mild COPD (*n* = 12) and severe COPD (*n = *10) and analyzed % cytotoxicity (**Fig. 5A**). Statistical analysis showed that CD56+ cells from subjects with severe COPD were significantly more cytotoxic than CD56+ cells from healthy smokers (*p* = 0.005). Furthermore, when subjects were stratified based on FEV_1_ % predicted, there was a significant inverse correlation with % cytotoxicity (**Fig. 5B**). Because circulating NK cells from peripheral blood of elderly subjects are reported to have decreased cytotoxic function [38], we analyzed the correlation between age and % cytotoxicity among lung CD56+ lymphocytes and found no significant correlation between the two variables (data not shown). Sixteen of the lung samples that were used for the cytotoxicity assay were also used for the phenotypic analyses in Figures 1 and 2. However, there was no correlation between the % cytotoxicity and the frequency of NK cells or CD56+ T cells or the percent of cells expressing CD16 or CD8. There was also no correlation between the cytolysis of target cells and ICS use.

In a separate set of analyses, we divided the subjects into two categorical groups: cytotoxic CD56+ lymphocytes and non-cytotoxic CD56+ lymphocytes. A given subject’s CD56+ lymphocytes were defined as cytotoxic if their observed cytotoxicity was greater than the geometric mean (8.5%). There were two subjects whose CD56+ lymphocytes met that definition, but whose CD8+ T cells and CD4+ T cells also had ≥8.5% cytotoxicity; because it was thus unclear whether their CD56+ lymphocytes were truly cytotoxic, or their results were artifactual, these subjects were removed from the analysis. We then looked for differences in pulmonary function, age, use of inhaled corticosteroids, pack-years, smoking status (active versus former), gender, and whether cancer was an indication for surgery between the subjects with non-cytotoxic CD56+ lymphocytes (*n* = 13) and those with cytotoxic CD56+ lymphocytes (*n* = 13). Both FEV_1_ % predicted (**Fig. 5C**) and DLCO % predicted (**Fig. 5D**) were significantly decreased in the subjects with cytotoxic CD56+ lymphocytes (*p* = 0.0002 and 0.009, respectively). Cancer as an indication for surgery was also significantly different between the groups (data not shown), as 12 out of the 13 subjects with non-cytotoxic CD56+ cells had lung resections due to cancer compared to only 4 out of the 13 subjects with cytotoxic CD56+ cells (*p* = 0.004). Importantly, however, these subjects also had significantly better lung function than those undergoing surgery for other indications. Subjects with non-cytotoxic CD56+ lymphocytes also tended to be older, although this observation did not reach statistical significance (*p* = 0.06, data not shown). Pack-years, smoking status, gender, and use of inhaled corticosteroids were not different between the two groups.

Finally, we performed multiple linear regression modeling to determine the ability of FEV_1_ % predicted, DLCO % predicted, age, or cancer as a surgical indication to independently predict whether a subject would have cytotoxic CD56+ cells. In a model incorporating those four variables (FEV_1_ % predicted and DLCO % predicted were first log-transformed), a higher FEV_1_ % predicted was associated with non-cytotoxic CD56+ cells (*p* = 0.05) **(** **Table 2** **)**. The other variables, DLCO % predicted, age, and cancer as an indication for surgery were not able to predict cytotoxicity as a categorical variable. This statistical model implies that the ability of CD56+ cells to kill autologous lung cells is significantly associated with spirometrically-defined COPD progression.

**Table 2:** Linear regression model to evaluate ability of variables to predict cytotoxicity of CD56+ cells.

Variable	B1	Std. error 1	Sig.	95% CI Lowerbound, Upper bound
Dependent variable = Cytotoxic(yes/no)				
Independent variables:				
Age	−0.12	0.01	0.34	−0.37, 0.01
FEV1, % predicted	1.66	0.81	0.05	−0.03, 3.34
DLCO, % predicted	−0.14	0.68	0.84	−1.54, 1.27
Resection for cancer	0.06	0.49	0.90	−0.95, 1.08

## Discussion

The results of this analysis of NK cells and CD56+ T cells from human lung tissue provide novel evidence that those CD56+ lymphocytes could contribute to emphysema progression by killing lung parenchymal cells, especially epithelial cells. Lung NK (CD3− CD56+) cells predominantly exhibited the differentiated (CD56^bright^ CD16+) phenotype associated with cytotoxicity rather than cytokine production [35]. Congruent with this phenotype, lung CD56+ cells were able to induce apoptosis in autologous lung parenchymal cells (defined as CD45−, high side scatter) without additional stimulation. Such natural cytotoxic activity was not seen in simultaneous analysis of purified CD8+ CD56− or CD4+ CD56− lung T cells from the same subjects. Killing was greater in COPD, whether defined categorically or by FEV_1_ % predicted, than in lung CD56+ cells isolated from subjects with normal spirometry, and was not explained in multiple regression analysis by age or cancer as the indication for surgery. These findings identify important differences in the mechanisms by which individual lung lymphocyte subsets contribute to specific COPD pathological phenotypes.

We assayed both populations of CD56+ lung lymphocytes together in our co-cultures experiments because we were primarily interested in contrasting NK activity with cytotoxicity by conventional CD8+ lung lymphocytes. Hence, in these studies we first removed all CD56+ cells using magnetic bead separation step. It is highly likely that the CD56+ fraction included some CD8+ cells, which may have contributed to the cytotoxicity of the CD56+ cells. We do not wish to imply that our CD8+ fraction contained all lung CD8+ cells, only that we assayed the CD56− CD8+ cells independently. When these experiments were being performed, we did not yet know whether it would be practical to next separate the CD56+ population into CD3− (NK) and CD3+ (CD56+ T cell) populations, because phenotypic characterization by flow cytometry were being performed simultaneously as yields from lung tissue permitted. The considerable variation we found in the ratios of these two CD56+ subsets between individual lung samples suggests that such a separation will need to be approached starting with physically larger lung samples devoted entirely to this purpose. This variability in ratios of CD56+ lymphocyte subsets also suggests that the proportion of autologous killing coming from the NK cell versus CD56+ T cell subset may vary between subjects. Although we did not detect a correlation between the percent cytotoxicity and the frequency of NK cells or CD56+ T cells among all lung lymphocytes or relative to each other, the subset of samples for which we had paired data was very likely under-powered to detect such relationships.

Our results build upon a variety of published data, as explained in the next four paragraphs. In particular, these findings refine and support the translational relevance of a cohesive, elegant series of studies from the Borchers laboratory that first linked cytotoxic T cell recognition of epithelial danger signals with emphysema [19], and which motivated our own recent murine experiments [10]. Borchers & colleagues showed that oxidative stress induced expression by cultured human airway epithelial cells of two groups of “danger signals”, the MICA & MICB surface receptors and the four UL-16 binding proteins. This change resulted from ERK-dependent translation of mRNA transcripts also present in healthy epithelial cells. Our observation that the percentage of CD326+ lung epithelial cells expressing an epitope common to MICA and MICB increased as FEV_1_ % predicted decreased complements their finding that MICA protein, quantified by immunoblot assay, was significantly increased in peripheral lung tissue from COPD patients [20]. Because these changes do not correlate with active smoking, the collective data of our two laboratories provide one plausible answer to the prevailing question of why immune-mediated damage in COPD progresses for years after smoking cessation. Both our results and theirs differ from another study that reported no difference between healthy smokers and COPD subjects in lung MICA expression, measured by immunohistochemistry [39]. The differences between techniques make it difficult to reconcile these three studies, which may have sampled subtly different anatomic regions.

Multiple epithelial cell danger signals including MICA, MICB and UL-16 binding proteins are recognized by NKG2D. That activating NK receptor is expressed by NK cell and CD8+ T cells, but importantly, has not been shown to induce cytoxicity by conventional CD8+ T cells. Our data also agrees with those of the Borchers laboratory [20] in showing no increase in NKG2D expression by lung cytotoxic lymphocytes in COPD, and significantly, we did not find a proportional increase in NK cells or CD56+ T cells in COPD, relative to smokers with preserved spirometry.

Thus, our collective data support a model in which a progressive increase in epithelial danger signals, rather than a change in the numbers or phenotype of cytotoxic lung lymphocytes, is associated with COPD progression. It might seem paradoxical that we did not detect expression by lung NK cells of NKp44. This receptor is known to be upregulated on activated NK cells [40], and the functional in vitro cytotoxicity by isolated CD56+ cells we found implies an activated phenotype. The likely explanation is that induction of surface expression of NKp44 is mainly associated with the CD56^bright^, CD16− subset of NK cells [41], which were rare in our human lung samples regardless of pulmonary function. Because we did not measure NKp44 expression following co-culture with the autologous lung cells, the possibility that it was upregulated only upon target cell recognition cannot be excluded.

However, one key way in which our data extend this line of investigation is by showing that only the CD56+ lung lymphocyte population, and specifically not conventional CD8+ CD56− lung T cells, induced rapid apoptosis of lung parenchymal cells. Identification of the responsible cell type was not possible in the design of previous studies [20]. Similarly, the demonstration in the current study of specific autologous parenchymal cell killing and its correlation with COPD, and not just the extension to parenchymal lung CD56+ cells, are the more significant ways in which we extend previous studies of NK cells in human sputum and bronchoalveolar lavage [8], [9]. By contrast, both previous studies used a classic NK target, the human K562 myeloid leukemia cell line, which lacks MHC Class I and is therefore highly susceptible to NK activity.

Although the current study is the first to show that human NK cells and CD56+ T cells induce apoptosis in autologous cells freshly isolated from the lungs, autologous killing by NK cells is itself well established. One example is killing of dendritic cells, despite their expression of normal levels of HLA Class I molecules [42], due to recognition of ‘altered self’ by the activating NK surface receptors NKp30 and NKp46 [43]. IL-2 activated NK cells lyse autologous bone marrow stromal cells via recognition by NKG2D of stromal cell damage, manifest by expression of MICA and ULBP3 [44]. In animal models of liver injury, NK cells kill autologous hepatocytes, stellate cells, and biliary epithelial cells [45]. Interestingly, using human biliary epithelial cells and autologous liver lymphocytes, Shimoda and colleagues demonstrated that unstimulated NK cells were not cytotoxic, but that specific combinations of TLR4 and TLR3 stimulation activated the NK cells to kill autologous biliary epithelial cells [46]. We have previously shown that CD56+ cells express TLRs and that the percentage of lung NK cells expressing TLR5, TLR6, and TLR2/1 correlated with worsening emphysema as determined by computed tomography quantification [29]. Although beyond the scope of this paper, future studies could explore a potential role for TLR stimulation in activating NK cell- or CD56+ T cell-mediated cytotoxicity.

It is essential to recognize that our findings do not exclude roles for conventional CD56− CD8+ T cells in emphysema progression. CD8+ T cells were initially linked to COPD because their numbers in human lung parenchyma and small airways correlate inversely with FEV_1_ [47]–[49]. It is unlikely that co-expression of CD8 by lung NK cells entirely confused the two cell types in these earlier single-color experiments; the direct correlation we found between co-expression of CD8 by lung NK cells and FEV_1_ % predicted implies that such misidentification would be lessened at greater degrees of COPD severity. However, CD8 co-expression on lung CD56+ cells might be a confounding factor in previous studies, including our own, if they were not specifically sought. Because the fraction of lung CD56+ cells among all lung lymphocytes was small in most subjects, and did not correlate with COPD status or severity, we believe it unlikely that failure to identify lung CD56+ CD8+ cells undermines seriously the broader literature on human lung conventional CD8+ T cells. Further studies would be need to test whether conventional lung CD8+ T cells might lyse targets in an MHC-dependent fashion; such assays typically require sever days of co-incubation and are thus not addressed by our assay system.

To our knowledge, there have been no published demonstrations of direct cytolysis of lung parenchymal cells by human lung CD8+ T cells, but there is strong support for their importance in development of emphysema from animal models. CD8 deficiency protects against emphysema in murine models of chronic CS or acrolein exposure [50], [51]. Although oligoclonal expansion of human lung CD8+ T cells was not identified in a study that did show such expansion of lung CD4+ T cells [52], oligoclonal expansion of CD8+ T cells did develop in CS-exposed mice and persists following cessation of exposure [53]. Adoptive transfer of CD3+ lung T cells from chronically CS-exposed mice induced lung inflammation in Rag2−/− mice, regardless of CS-exposure of the recipients [54]. CD8+ T cells have considerable potential to damage lung parenchyma, which they could do directly or indirectly. We have previously shown that in unstimulated human lung CD8+ T cells, mRNA transcripts for IFN-γ, perforin and granzyme B correlate inversely to FEV_1_ % predicted [27], [28]. Production by lung CD8+ T cells of IFN-γ and chemokines such as CXCL10 can induce production of elastinolytic matrix metalloproteinases by lung macrophages [55]–[57]. Additionally, although lung CD56− CD8+ T cells did not spontaneously kill lung parenchymal cells in the current study, at least over the time-frame we tested, their direct cytolytic activity might still drive emphysema episodically when they recognize viral antigens during COPD exacerbations or pneumonias [58].

There are several limitations to this study. The first, to which we have already alluded, is uncertainty on the balance between the two CD56+ lung effector cell types (NK cells vs. CD56+ T cells) in the cytotoxicity assay. We suspect that net NK activity results from CD3− NK cells in most subjects, but as stated above, the balance may vary between individuals. Secondly, we are unable to identify with total certainty the cell type being targeted for killing. We hypothesize that they are primarily epithelial cells, as we gated on high side-scatter CD45− cells, mimicking the strategy for brushing-derived airway epithelial cells utilized by Hodge et al. [37]. The inverse correlations of FEV_1_ % predicted with both expression of MICA/MICB by cells expressing the epithelial cell-specific marker CD326 and with specific killing would be compatible with that possibility, but clearly do not prove it. There are several technical factors that make target cell identification technically challenging and well-beyond the scope of the current study. Many markers of cell lineage are intracellular and thus are unsuited to pair with analysis of apoptosis by simultaneous annexin-V/7-AAD staining, which we chose for its high sensitivity and lack of reliance on radioactivity. Moreover, surface receptors of cell lineage might be lost during the substantial changes accompanying apoptosis. A further limitation is that the flow cytometric analyses of NKG2D and MICA/MICB were performed on a separate cohort of subjects, making it impossible to correlate the expression of these receptors or ligands with cytotoxicity. Because these experiments depend on human lung tissue removed exclusively for clinical indications, it is not always possible to assure complete matching between smokers with COPD versus those without for all clinical variables, nor do we currently have access to viable lung tissue from never-smokers. We have attempted to control for those variables in our linear regression model, but it remains an unavoidable limitation.

In summary, human lung CD56+ lymphocytes, and not conventional CD56− CD8+ or CD56− CD4+ T cells, induced apoptosis of CD45− autologous lung parenchymal cells in culture without additional stimulation. Because this CD56+ cytotoxicity was aggravated in severe COPD, it could plausibly contribute to progression of emphysema. As shown here and by others [20], this trend in cytotoxicity appears to be driven by steadily increased expression by lung epithelial cells of danger signals (including but likely not limited to the NKG2D ligands MICA and MICB), rather than by increased expression by lung CD56+ lymphocytes of NKG2D (or other receptors we tested). Such efficient spontaneous auto-aggressive behavior by innate lung lymphocytes provides an explanation for lung destruction in COPD that is alternative, although not mutually exclusive, to models of clonally-specific adaptive autoimmunity.
